# Human leukocyte antigens are associated with salivary level of active MMP‐8

**DOI:** 10.1002/cre2.419

**Published:** 2021-08-27

**Authors:** Matti Mauramo, Elina Mauramo, Timo Sorsa, Taina Tervahartiala, Ismo T. Räisänen, Tuomas Waltimo

**Affiliations:** ^1^ Department of Pathology University of Helsinki and Helsinki University Central Hospital Helsinki Finland; ^2^ Department for Oral Health and Medicine and Center of Salivary Diagnostics and Hyposalivation UZB University Centre for Dental Medicine Basel, University of Basel Basel Switzerland; ^3^ Department of Public Health University of Helsinki Helsinki Finland; ^4^ Department of Oral and Maxillofacial Diseases Helsinki University Central Hospital, Institute of Dentistry Helsinki Finland; ^5^ Department of Oral Diseases Karolinska Institutet Huddinge Sweden

**Keywords:** human leukocyte antigen, mmp8, periodontitis, salivary

## Abstract

**Objectives:**

This case control study examined the associations of HLA antigens and periodontitis with the salivary level of active MMP‐8 (aMMP‐8).

**Materials and Methods:**

A total of 202 subjects, registered as Swiss bone marrow donors, participated in the study. HLA‐A, ‐B, and ‐C types were determined by serology or PCR. Saliva samples were collected from subjects, followed by a periodontal examination. The salivary level of aMMP‐8 was determined with immunofluorometric assay.

**Results:**

The mean salivary level of aMMP‐8 was directly comparable to the grade of periodontitis and increased from healthy to mild/moderate to severe (125.0 ± 132.1, 200.6 ± 170.2, 290.1 ± 202.3 ng/ml; *p* < 0.001 between each group, respectively). The only association between the HLA types and the salivary level of aMMP‐8 was observed in subjects with HLA‐A11. Subjects with healthy periodontium and HLA‐A11 had a lower level of aMMP‐8 (49.2 ± 32.5 ng/ml) compared with subjects without HLA‐A11 (123.6 ± 119.2; *p* = 0.048). Among subjects with periodontitis, a higher level of aMMP‐8 (394.2 ± 255.6 ng/ml) was observed in subjects with HLA‐A11 compared with subjects without HLA‐A11 (201.1 ± 146.1 ng/ml; *p* < 0.002). This finding was statistically significant also after adjusting for sex, age, smoking, tooth brushing and the number of medications (*p* < 0.05).

**Conclusions:**

HLA‐A11 is associated with the salivary level of aMMP‐8 which contributes to the subject's immune and inflammatory response in periodontium.

## 1 INTRODUCTION

Oral bacteria, and periodontal pathogens in particular, induce inflammation in the gingiva. This may lead to irreversible attachment loss of a tooth, that is, periodontitis. Despite the frequent occurrence of periodontal pathogens among normal population, the degree and the development of periodontal disease vary from a completely healthy periodontium to mild, slowly progressing periodontitis and even to necrotizing periodontitis (Tonetti et al., 2018). Thus, in the pathogenesis of periodontitis, synergistic effects of various causative factors–such as lifestyle factors including smoking and oral healthcare habits, as well as comorbidities for example, diabetes–have been postulated (Loos & Van Dyke, 2020). Moreover, the susceptibility to periodontitis has been observed to be partly determined by a genetic predisposition (Loos & Van Dyke, 2020).

Recognition of the antigens of periodontal pathogens is crucial for an immune response. The binding of peptide fragments of foreign proteins for presentation to antigen‐specific T cells is the principal function of the major histocompatibility complex also known as the human leukocyte antigen (HLA) complex (Kumar et al., 2018). The HLA system is highly pleomorphic. Each individual inherits one HLA allele from each parent and typically expresses two different molecules of every loci which fall into two major classes: Class I molecules (HLA‐A, HLA‐B, and HLA‐C) expressed on virtually all nucleated cells and Class II molecules (HLA‐DP, ‐DQ, and ‐DR) expressed mainly on antigen presenting cells, namely dendritic cells, macrophages, Langerhans cells and B cells (Kumar et al., 2018). In addition to the antigen presenting cells, also other cells, including endothelial cells, fibroblasts, and possibly neutrophils can be induced to express Class II molecules (Vono et al., 2017). Since the 1970s, studies have been conducted to determine the associations of HLA Classes I and II antigens with periodontitis, and several associations of HLA alleles with different forms of periodontitis have already been observed. At least HLA‐A9, −A11, −A29, −B14, −B15, and −DR4 have been observed to be associated with susceptibility for periodontitis, whereas HLA‐A2 and –B5 are potentially protective against periodontitis (Machulla et al., 2002; Stein et al., 2008).

Many inflammatory cells secrete matrix metalloproteinases (MMPs). In periodontitis, genetically distinct but structurally related MMPs degrade almost all extracellular matrix components. Elevated levels of active collagenase MMP‐8 (aMMP‐8) in particular have been shown to be associated with periodontitis and accelerated disease progression (Al‐Majid et al., 2018; Sorsa et al., 2020). It is noteworthy, that especially aMMP‐8 but not total or latent MMP‐8 has been implicated as a successful biomarker for periodontitis (Sorsa et al., 2020; Tonetti et al., 2018). Thus, an aMMP‐8 point‐of‐care/chair‐side test has recently been developed for diagnosing and monitoring periodontal diseases and therapy as well as for identifying genetic susceptibility to periodontitis (Alassiri et al., 2018; Heikkinen et al., 2016; Rathnayake et al., 2017; Sorsa et al., 2020). In addition to periodontitis, recent studies have shown aMMP‐8 to be associated with chronic inflammation and inflammatory diseases including obesity, rheumatoid arthritis and cardiovascular diseases (Äyräväinen et al., 2018; Lauhio et al., 2016; Tuomainen et al., 2014). Furthermore, aMMP‐8 has been observed to be associated with an elevated C‐reactive protein level among subjects with the diabetes risk genotype HLA‐DQ2/8 (Odermarsky et al., 2018).

To date, this is the first study that aims to examine the associations of the salivary level of aMMP‐8 with HLA Class I (HLA‐A and −B) and Class II (HLA–DRB) alleles among subjects with or without periodontitis. Furthermore, the study aims to confirm and further extend from the previous findings concerning the associations of the salivary level of aMMP‐8 with periodontitis in a normal population volunteered and listed in Swiss bone marrow registry. The hypothesis is that the HLA types previously shown to be related to periodontitis are also associated with salivary aMMP‐8.

## 2 MATERIALS AND METHODS

### 2.1 Ethical consideration

This prospective case–control study was approved by the Ethics Committee of Basel, Switzerland (Ethikkommission beider Basel, Ref. Nr. EK:357/08). The study has been conducted according to the principles expressed in the Declaration of Helsinki, 1975, and a written informed consent was obtained from each subject.

### 2.2 Study subjects and HLA typing

The study subjects were recruited from the Swiss bone marrow donor registry by sending an invitation letter once to the subjects randomly selected from the register of The Blood Transfusion Service SRC Basel, Switzerland. All adult (>18 years) subjects who volunteered to participate in the study were included, resulting in a total of 202 subjects. None of the volunteers were excluded by the predefined criteria of congenital or acquired heart valve diseases or high‐dose immunosuppressive treatments that would have been indications for antibiotic prophylaxis prior to the clinical examination. For the bone marrow donor register, the HLA types of the participants were already determined as follows: 1. HLA‐A and ‐B types were determined by serology, that is, by microlymphocytotoxicity using the Biotest AB120 trays (Biotest AG, Dreieich, Germany). 2. HLA‐DRB1 types were obtained by PCR‐sequence‐specific oligonucleotide probe hybridization using commercial reagents (bioMérieux, Lyon, and One Lambda, Ingen, France). Because the majority of donors included in the study had only been tested by DRB1 generic typing, only low resolution level data (two digits) were considered in the present analysis.

### 2.3 Clinical oral examinations

Anamnestic data collected for this study included tobacco smoking (current, former, never), the use of medications (*n*) and oral hygiene habits (tooth brushing frequency: >1 times/day, once/day, <once/day).

The clinical oral examinations of the patients were carried out by M. M. between 2008 and 2010. Prior to any other procedures, stimulated whole saliva was collected as previously described (Mauramo et al., 2014). Thereafter, a complete clinical periodontal status including probing pocket depth and clinical attachment level measurement (CAL) from six sites/tooth was performed with a pressure calibrated periodontal probe. Periodontitis was classified to severe, moderate, mild or no periodontitis according to the Center for Disease Control and Prevention and the American Academy of Periodontology (Eke et al., 2012; Tonetti et al., 2018). Briefly, periodontitis was diagnosed severe if ≥2 interproximal sites with CAL ≥6 mm and ≥ 1 interproximal sites with PPD ≥5 mm; moderate if ≥2 interproximal sites with CAL ≥4 mm or ≥ 2 interproximal sites with PPD ≥5 mm; and mild if ≥2 interproximal sites with CAL ≥3 mm and ≥ 2 interproximal sites with PPD ≥4 mm or ≥ 1 interproximal sites with PPD ≥5 mm.

### 2.4 aMMP‐8 analysis

The mean salivary level of aMMP‐8 was determined with time‐resolved immunofluorometric assay as previously described (Hanemaaijer et al., 1997; Heikkinen et al., 2010). The monoclonal MMP‐8‐specific antibodies 8708 and 8706 (Medix Biochemica Oy Ab, Espoo, Finland) were used as a catching and tracer antibody, respectively. Europium chelate was used to label the tracer antibody. The samples were diluted in assay buffer containing 20 mM Tris–HCl, pH 7.5; 0.5 M NaCl; 5 mM CaCl2; 50 μM ZnCl2; 0.5% bovine serum albumin; 0.05% sodium azide; and 20 mg/L diethylenetriamine pentaacetic acid and incubated for 1 h followed by incubation for 1 h with the tracer antibody. Enhancement solution was added, and after 5 min, the fluorescence was measured (1234 Delfia Research Fluoremeter; Wallac, Turku, Finland), and the levels of aMMP‐8 were expressed as microgram per liter (mg/L).

### 2.5 Statistical analysis

Subjects were grouped according to periodontal health: 1. healthy periodontium or 2. periodontitis which was further divided according to the grade of periodontitis. Grade “mild” (*n* = 5) was combined with grade “moderate” periodontitis. Differences in the descriptives between subjects with healthy periodontium compared with subjects with periodontitis were statistically tested (*t*‐test or chi‐square). The association of the mean salivary level of aMMP‐8 with periodontitis was determined with non‐parametric Kruskal–Wallis tests. In the next phase, the mean salivary level of aMMP‐8 was compared separately within the healthy periodontium group and the periodontitis group between subjects with or without a certain HLA‐A, ‐B or –DRB allele (Mann–Whitney *U* test). Furthermore, to adjust for confounding factors (sex, age, tobacco smoking, number of medications and oral hygiene habits), a separate multivariate general linear model (ANCOVA) was conducted. *p*‐value of <0.05 was considered as statistically significant. SPSS (version 25; IBM) was used for conducting the statistical analyses.

## 3 RESULTS

### 3.1 Salivary level of aMMP‐8 and periodontitis

Of the 202 subjects, 86 subjects had a healthy periodontium and 116 subjects had periodontitis of which 83 had mild or moderate and 33 severe grades of periodontitis. Sex, age, the number of medications, the most frequent/common? diseases, smoking and tooth brushing frequency of the subjects according to periodontium health are presented in Table 1. In general, subjects with a healthy periodontium were younger (mean age: 42.9 vs. 48.2 years, *p* < 0.001) and more often female (68.6% vs. 52.6%, *p* = 0.03) compared with subjects with periodontitis, respectively. There was also a statistically significant difference in the prevalence of self‐reported asthma (16.3% vs. 6.0%, *p* = 0.02) and hypertension (3.5% vs. 15.5%, *p* = .009) between the subjects with a healthy periodontium compared with subjects with periodontitis.

**TABLE 1:** Descriptives of the subjects with healthy periodontium and subjects with periodontitis

		Healthy (*n* = 86)	Periodontitis (*n* = 116)
Sex	Male, *n* (%)	27 (31.4)	55 (47.4)
	Female, *n* (%)	59 (68.6)	61 (52.6)
Age	Mean (range)	42.9 (25–57)*	48.2 (29–56)*
Number of medications	Mean (range)	0.3 (0–2)	0.5 (0–5)
Astma	*n* (%)	14 (16.3)*	7 (6.0)*
Diabetes (T2)	*n* (%)	0	3 (2.6)
Hypertension	*n* (%)	3 (3.5)*	18 (15.5)*
Reumatic dis.	*n* (%)	2 (2.3)	2 (1.7)
Smoking	Never, *n* (%)	52 (60.5)	58 (50.0)
	Former, *n* (%)	21 (24.4)	38 (32.8)
	Current, *n* (%)	13 (15.1)	21 (17.2)
Tooth brushing	>1 times/day, *n* (%)	73 (84.9)	97 (83.6)
	1 times/day, *n* (%)	12 (14.0)	20 (16.4)
	<1times/day, *n* (%)	1 (1.1)	—
Periodontitis grade	Mild, *n* (%)	—	5 (4.3)
	Moderate, *n* (%)	—	79 (67.5)
	Severe, *n* (%)	—	33 (28.2)

* *p* < 0.05.

**FIGURE 1:**
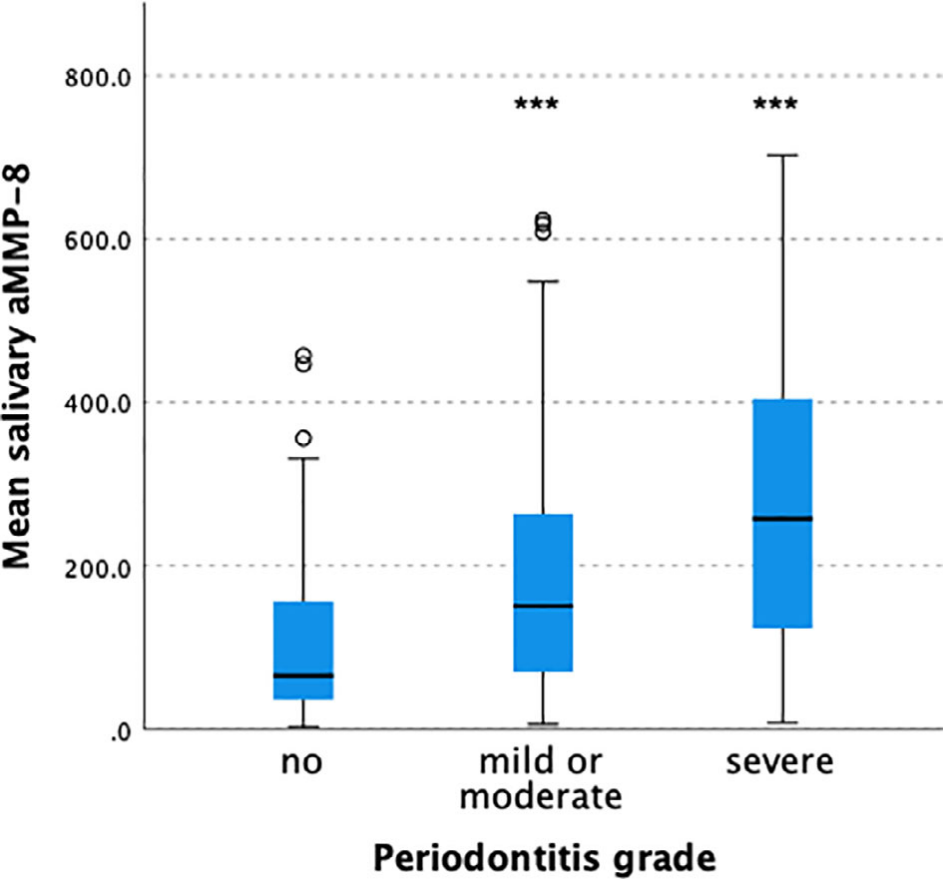
The salivary levels of active collagenase matrix metalloproteinases‐8 (aMMP‐8) according to grade of periodontitis. The box plot represents mean, first and third quartiles, and maximum and minimum values, with the exceptions of outliers (circles: third quartile +1.5*interquartile range); ***correspond to *p* < 0.001 (healthy vs. mild/moderate or severe grades of periodontitis)

The salivary level of active MMP‐8 was statistically significantly associated with periodontitis. The mean salivary level of aMMP‐8 increased according to the grade of periodontitis from healthy (125.0 ± 132.1 ng/ml) via mild or moderate periodontitis (200.6 ± 170.2 ng/ml) to severe periodontitis (290.1 ± 202.3 ng/ml); the *p*‐value between groups <0.001. Figure 1.

### 3.2 Associations of HLA types with aMMP‐8

**FIGURE 2:**
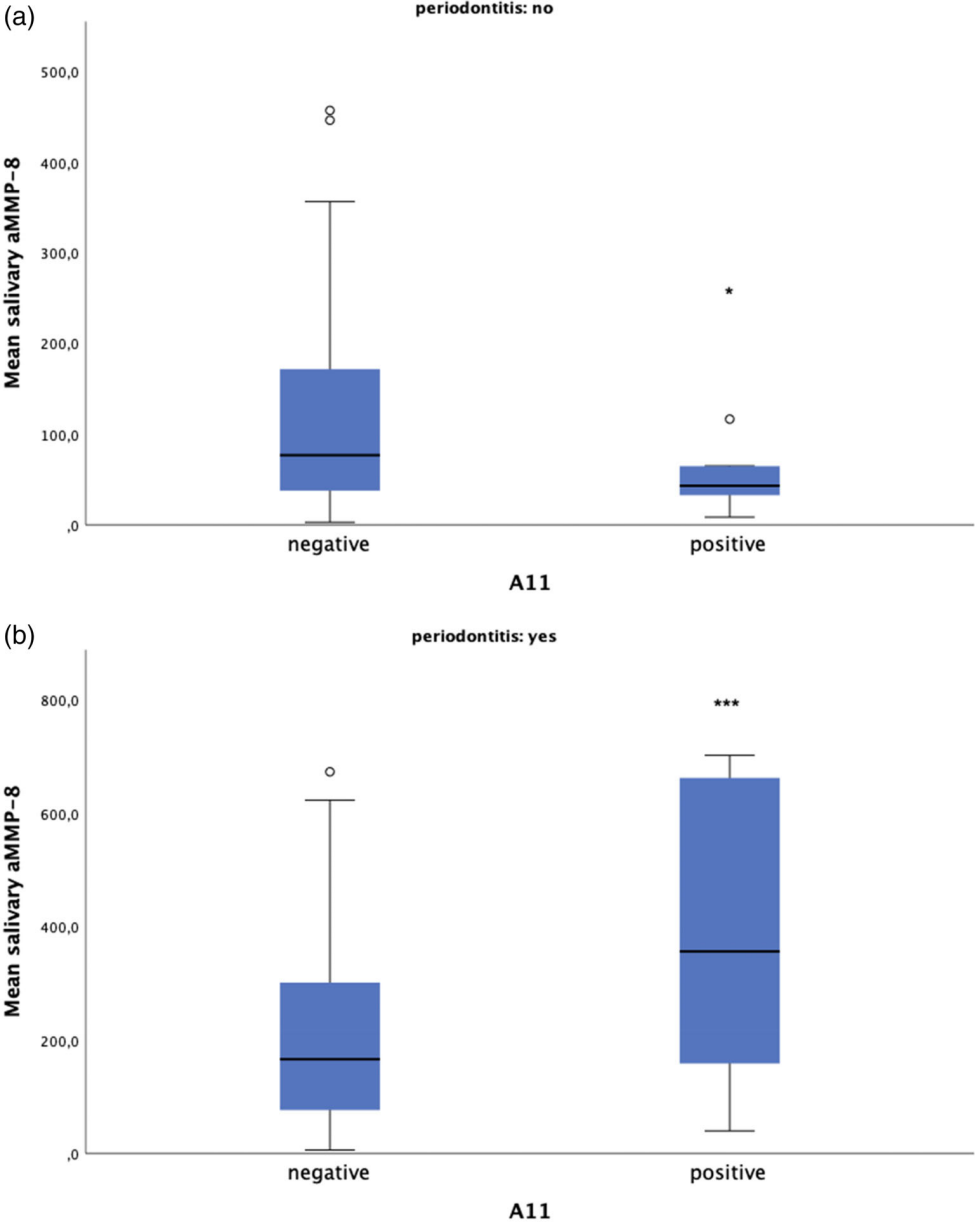
The salivary levels of active collagenase matrix metalloproteinases‐8 (aMMP‐8) in subjects with or without periodontitis and HLA‐A11. The box plot represents mean, first and third quartiles, and maximum and minimum values, with the exceptions of outliers (circles: third quartile +1.5*interquartile range); *correspond to *p* < 0.05 and ***correspond to *p* < 0.001

Associations of HLA Classes I and II with the mean salivary level of aMMP‐8 were analyzed separately according to the health of periodontium (healthy periodontium or periodontitis). Thirteen HLA‐A, 17 HLA‐B, and 10 HLA‐DR types were included in the analysis (Supplementary Tables 1, 2 and 3). Contrasting findings concerning the association between HLA‐A11 and periodontal status were observed. Subjects with a healthy periodontium and HLA‐A11 had a lower mean salivary level of aMMP‐8 (49.2 ± 32.5 ng/ml) compared to subjects without HLA‐A11 (123.6 ± 119.2; *p* = 0.048). However, subjects with periodontitis and HLA‐A11 had a higher mean salivary level of aMMP‐8 (394.2 ± 255.6 ng/ml) compared to subjects without HLA‐A11 (201.1 ± 146.1 ng/ml; *p* = 0.002). Figure 2.

In the general linear model, the association of HLA‐A11 with the salivary level of aMMP‐8 remained statistically significant (*p* < 0.05) among subjects with periodontitis when sex, age, smoking, tooth brushing and the number of medications were adjusted for (Estimated marginal means: HLA‐A11 neg: 200.7, 95%CI: 168.3–233.1; HLA pos: 398.1, 95%CI: 287.1–509.1). However, after adjusting for confounders, the association of HLA‐A11 with aMMP‐8 among subjects with healthy periodontium was no more statistically significant.

HLA‐B and HLA‐DRB types had no associations with the salivary level of aMMP‐8 in subjects with healthy periodontium or periodontitis (all *p*‐values >0.05; Supplementary Tables 2 and 3).

## 4 DISCUSSION

Several important associations of HLA types with inflammatory diseases have been confirmed over the last decades. HLA alleles have been shown to contribute to the pathogenesis of type 1 diabetes, multiple sclerosis, celiac diseases, systemic lupus erythematosus, rheumatoid arthritis and spondyloarthritis, among others (Kumar et al., 2018). Several attempts have also been made to reveal associations of HLA types with periodontitis, and several different HLA types have been shown to increase susceptibility for periodontitis (Stein et al., 2008). This is the first study on the associations of HLA types with the salivary level of active MMP‐8 in periodontitis. HLA‐A11 was associated with a higher salivary level of aMMP‐8 in subjects with periodontitis. In line with the hypothesis and the results of this study, HLA‐A11 allele has been observed to be more common among a comparable population of German subjects with rapidly progressive and adult periodontitis (Machulla et al., 2002).

Of the various inflammatory proteins present in saliva, aMMP‐8 has been shown to be a specific and sensitive biomarker of periodontitis, and has been implicated to be used as a biomarker of the disease in the new periodontitis disease classification (Alassiri et al., 2018; Al‐Majid et al., 2018; Rathnayake et al., 2017; Sorsa et al., 2020; Tonetti et al., 2018). In keeping with previous results, also in the current study, the salivary level of active MMP‐8 was clearly associated with periodontitis and its grade. The mean salivary level of aMMP‐8 increased according to the periodontitis grade from healthy to mild or moderate and to severe periodontitis. In this study, subjects with a healthy periodontium where younger and more often female. This is in line with several epidemiological studies which have demonstrated periodontitis to be more common among male and elderly people (Eke et al., 2015). Furthermore, MMP‐8 has been shown to be associated with periodontitis among both sexes and even in pediatric population, and thus, these differences between the groups are as expected, and insignificant in this context (Alfant et al., 2008).

The pathogenesis of periodontitis is a process with acute and chronic phases and overlapping and simultaneous effects of innate and acquired immunity. Acquired immunity, in particular, relies on class II HLA antigens expressed on antigen presenting cells and T‐ and B‐lymphocytes, whereas in acute inflammation polymorphonuclear leukocytes, possessing mainly class I HLA antigens, are particularly abundant. In line with this, both Classes I and II HLA antigens have been shown to be associated with periodontitis (Stein et al., 2008). MMP‐8, also known as neutrophil collagenase, is assumed to be released mainly by polymorphonuclear leukocytes for example, neutrophils during an acute phase of inflammation. However, fibroblasts, endothelial cells and monocytes/macrophages can also express and produce MMP‐8 (Herman et al., 2001; Wen et al., 2015). Furthermore, monocytes which contribute mainly to innate immune response, have been shown to overexpress HLA‐DR molecules in periodontitis (Almubarak et al., 2020). However, this study could not demonstrate any associations of HLA‐B and HLA‐DRB types with the salivary level of aMMP‐8 among subjects with or without periodontitis. This might reflect the multicausal pathophysiology of periodontitis with overlapping etiological factors, including chronic inflammatory diseases, smoking, direct bacterial insults and a variety of host derived pro‐inflammatory cytokines (e.g., interleukin‐17, interferon‐gamma, tumor necrosis factor) and proteinases (Slots, 2017).

Periodontitis and other chronic inflammatory diseases are known to share similarities in their pathogenic features and genetic predisposition. In particular, rheumatoid arthritis (RA) and periodontitis are frequently associated with each other and they share similar inflammatory and tissue destruction processes (Ceccarelli et al., 2019). Furthermore, both RA and periodontitis have been shown to be associated with HLA‐DRB*1 (Ceccarelli et al., 2019). In subjects with RA, the serum level of aMMP‐8 has been shown to be elevated, similarly to what is observed in subjects with periodontitis (Äyräväinen et al., 2018; Mirrielees et al., 2010). Patients with RA and periodontitis have been shown to exhibit increased levels of aMMP‐8 in gingival crevicular fluid compared with systemically healthy subjects with periodontitis (Kirchner et al., 2017). In this study, only four subjects had a self‐reported rheumatic disease and they were equally distributed according to the state of periodontium: two with healthy periodontium and two with periodontitis. The mean salivary level of aMMP‐8 was higher among the two subjects with rheumatic disease and periodontitis compared with the two with rheumatic disease and healthy periodontium (382.7 vs. 75.0 ng/ml). However, within the limits of this study, the effect of rheumatic disease on the salivary MMP‐8 level could not be adequately studied and more extensive studies are needed to confirm the previous results.

Particularly interesting is that studies have shown the frequency of HLA‐A11 allele to be more common in subjects with rheumatoid arthritis and non‐specific polyarthritis (Bhatia et al., 1988; Tsuchiya et al., 2001; Vaterlaws et al., 1989). In contrast, HLA‐A11 has been observed to be less common in subjects with psoriatic arthritis compared with healthy controls (Schneeberger et al., 2015). In this study, HLA‐A11 frequency was different in relation to the mean salivary level of aMMP‐8 among subjects with or without periodontitis. In subjects with periodontitis, a higher level of salivary aMMP‐8 was observed among subjects with HLA‐A11, whereas in subjects with healthy periodontium, a lower level of salivary aMMP‐8 was observed among subjects with HLA‐A11. However, the latter association of HLA‐A11 with the salivary level of aMMP‐8 among subjects with healthy periodontium was only borderline statistically significant (*p* = 0.048), and lost the statistical significance when confounding factors were adjusted for. However, the differing association of HLA‐A11 with the salivary level of aMMP‐8 according to the health of the periodontium, as well as the differing association of HLA‐A11 with different forms of arthrosis (rheumatoid vs. psoriatic) are peculiar and might be partly explained by concurrent effects of HLA class I and II molecules. In accordance with this hypothesis, a previous study has shown HLA‐A11 to be associated with a more severe phenotype of RA if in combination with DRB1*0405 (Tsuchiya et al., 2001). A limitation of this study is that HLA polymorphism in an unselected population results in a wide variety of HLA types, and thus the associations of different combinations of different HLA types could not be studied with this number of subjects. Only three subjects with periodontitis had the combination of HLA‐A11 and DRB1*04. In this small group of subjects, the salivary level of aMMP‐8 was high (mean 494 ng/ml), but proper assessment of HLA types in combinations warrants further studies with a higher number of subjects and/or with participants pre‐selected according to HLA types.

In conclusion, this is the first study to demonstrate HLA‐A11 to be associated with an elevated level of aMMP‐8, predisposing subjects to periodontitis. This study also confirms the previous studies showing that the salivary aMMP‐8 level reflects the grade of periodontitis and can be used as an adjunctive diagnostic tool and biomarker to assess periodontitis.

## CONFLICT OF INTEREST

The authors declare no conflict of interest. Prof. Timo Sorsa is an inventor of US‐patents 5652223, 5736341, 5866432 and 614347.

## Supporting information


**Table S1** Supporting information

## Data Availability

The data that support the findings of this study are available from the corresponding author upon reasonable request.

## References

[cre2419-bib-0001] Alassiri, S. , Parnanen, P. , Rathnayake, N. , Johannsen, G. , Heikkinen, A. M. , Lazzara, R. , Schoor, P. , Schoor, J. G. , Tervahartaila, T. , Gieselmann, D. , & Sorsa, T. (2018). The ability of quantitative, specific, and sensitive point‐of‐care/chair‐side Oral fluid Immunotests for aMMP‐8 to detect periodontal and Peri‐implant diseases. Disease Markers, 2018, 1306396. 10.1155/2018/1306396 30154936 PMC6098860

[cre2419-bib-0002] Alfant, B. , Shaddox, L. M. , Tobler, J. , Magnusson, I. , Aukhil, I. , & Walker, C. J. (2008). Matrix metalloproteinase levels in children with aggressive periodontitis. Periodontology, 79(5), 119–126. 10.1902/jop.2008.070513 18454660

[cre2419-bib-0003] Al‐Majid, A. , Alassiri, S. , Rathnayake, N. , Tervahartiala, T. , Gieselmann, D. R. , & Sorsa, T. (2018). Matrix metalloproteinase‐8 as an inflammatory and prevention biomarker in periodontal and peri‐implant diseases. International Journal of Dentistry, 16, 7891323. 10.1155/2018/7891323 PMC6165625 30305812

[cre2419-bib-0004] Almubarak, A. , Tanagala, K. , Papapanou, P. N. , Lalla, E. , & Momen‐Heravi, F. (2020). Disruption of monocyte and macrophage homeostasis in periodontitis. Frontiers in Immunology, 11, 330. 10.3389/fimmu.2020.00330 32210958 PMC7067288

[cre2419-bib-0005] Äyräväinen, L. , Heikkinen, A. M. , Kuuliala, A. , Ahola, K. , Koivuniemi, R. , Laasonen, L. , Moilanen, E. , Hämäläinen, M. , Tervahartiala, T. , Meurman, J. H. , Leirisalo‐Repo, M. , & Sorsa, T. (2018). Inflammatory biomarkers in saliva and serum of patients with rheumatoid arthritis with respect to periodontal status. Annals of Medicine, 50(4), 333–344. 10.1080/07853890.2018.1468922 29683364

[cre2419-bib-0006] Bhatia, K. , Richens, J. , Prasad, M. L. , & Koki, G. (1988). High prevalence of the haplotype HLA‐A11, B27 in arthritis patients from the highlands of Papua New Guinea. Tissue Antigens, 31(2), 103–106. 10.1111/j.1399-0039.1988.tb02071.x 3259735

[cre2419-bib-0007] Ceccarelli, F. , Saccucci, M. , Di Carlo, G. , Lucchetti, R. , Pilloni, A. , Pranno, N. , Luzzi, V. , Valesini, G. , Polimeni, A. (2019). Periodontitis and rheumatoid arthritis: The same inflammatory mediators?. Mediators of Inflammation, 6034546. 10.1155/2019/6034546, 1, 8 PMC6525860 31191116

[cre2419-bib-0008] Eke, P. I. , Dye, B. A. , Wei, L. , Slade, G. D. , Thornton‐Evans, G. O. , Borgnakke, W. S. , Taylor, G. W. , Page, R. C. , Beck, J. D. , & Genco, R. J. (2015). Update on prevalence of periodontitis in adults in the United States: NHANES 2009 to 2012. Journal of Periodontology, 86(5), 611–622. 10.1902/jop.2015.140520 25688694 PMC4460825

[cre2419-bib-0009] Eke, P. I. , Page, R. C. , Wei, L. , Thornton‐Evans, G. , & Genco, R. J. (2012). Update of the case definitions for population‐based surveillance of periodontitis. Journal of Periodontology, 83(12), 1449–1454. 10.1902/jop.2012.110664 22420873 PMC6005373

[cre2419-bib-0010] Hanemaaijer, R. , Sorsa, T. , Konttinen, Y. T. , Ding, Y. , Sutinen, M. , Visser, H. , Van Hinsbergh, V. W. M. , Helaakoski, T. , Kainulainen, T. , Rönkä, H. , Tschesche, H. , & Salo, T. (1997). Matrix metalloproteinase‐8 is expressed in rheumatoid synovial fibroblasts and endothelial cells. Regulation by tumor necrosis factor‐alpha and doxycycline. The Journal of Biological Chemistry, 272(50), 31504–31509. 10.1074/jbc.272.50.31504 9395486

[cre2419-bib-0011] Heikkinen, A. M. , Nwhator, S. O. , Rathnayake, N. , Mäntylä, P. , Vatanen, P. , & Sorsa, T. (2016). Pilot study on Oral health status as assessed by an active matrix Metalloproteinase‐8 Chairside Mouthrinse test in adolescents. Journal of Periodontology, 87(1), 36–40. 10.1902/jop.2015.150377 26430926

[cre2419-bib-0012] Heikkinen, A. M. , Sorsa, T. , Pitkäniemi, J. , Tervahartiala, T. , Kari, K. , Broms, U. , Koskenvuo, M. , & Meurman, J. H. (2010). Smoking affects diagnostic salivary periodontal disease biomarker levels in adolescents. Journal of Periodontology, 81(9), 1299–1307. 10.1902/jop.2010.090608 20450405

[cre2419-bib-0013] Herman, M. P. , Sukhova, G. K. , Libby, P. , Gerdes, N. , Tang, N. , Horton, D. B. , Kilbride, M. , Breitbart, R. E. , Chun, M. , & Schönbeck, U. (2001). Expression of neutrophil collagenase (matrix metalloproteinase‐8) in human atheroma: A novel collagenolytic pathway suggested by transcriptional profiling. Circulation, 104(16), 1899–1904. 10.1161/hc4101.097419 11602491

[cre2419-bib-0014] Kirchner, A. , Jäger, J. , Krohn‐Grimberghe, B. , Patschan, S. , Kottmann, T. , Schmalz, G. , Mausberg, R. F. , Haak, R. , & Ziebolz, D. (2017). Active matrix metalloproteinase‐8 and periodontal bacteria depending on periodontal status in patients with rheumatoid arthritis. Journal of Periodontal Research, 52(4), 745–754. 10.1111/jre.12443 28321852

[cre2419-bib-0015] Kumar, V. , Abbas, A. K. , & Aster, J. (2018). Robbins Basic Pathology (10th ed., pp. 130–162). New York, NY: Elsevier.

[cre2419-bib-0016] Lauhio, A. , Färkkilä, E. , Pietiläinen, K. H. , Åström, P. , Winkelmann, A. , Tervahartiala, T. , Pirilä, E. , Rissanen, A. , Kaprio, J. , Sorsa, T. A. , & Salo, T. (2016). Association of MMP‐8 with obesity, smoking and insulin resistance. European Journal of Clinical Investigation, 46(9), 757–765. 10.1111/eci.12649 27296149

[cre2419-bib-0017] Loos, B. G. , & Van Dyke, T. E. (2020). The role of inflammation and genetics in periodontal disease. Periodontology 2000, 83(1), 26–39. 10.1111/prd.12297 32385877 PMC7319430

[cre2419-bib-0018] Machulla, H. K. , Stein, J. , Gautsch, A. , Langner, J. , Schaller, H. G. , & Reichert, S. (2002). HLA‐A, B, Cw, DRB1, DRB3/4/5, DQB1 in German patients suffering from rapidly progressive periodontitis (RPP) and adult periodontitis (AP). Journal of Clinical Periodontology, 29(6), 573–579. 10.1034/j.1600-051x.2002.290614.x 12296785

[cre2419-bib-0019] Mauramo, M. , Ramseier, A. M. , Buser, A. , Tiercy, J. M. , Weiger, R. , & Waltimo, T. (2014). Associations of HLA‐A, ‐B and ‐DRB1 types with oral diseases in Swiss adults. PLoS One, 9(7), e103527. 10.1371/journal.pone.0103527 25072155 PMC4114782

[cre2419-bib-0020] Mirrielees, J. , Crofford, L. J. , Lin, Y. , Kryscio, R. J. , Dawson, D. R., 3rd , Ebersole, J. L. , & Miller, C. S. (2010). Rheumatoid arthritis and salivary biomarkers of periodontal disease. Journal of Clinical Periodontology, 37(12), 1068–1074. 10.1111/j.1600-051X.2010.01625.x 20880053 PMC2980566

[cre2419-bib-0021] Odermarsky, M. , Pesonen, E. , Sorsa, T. , Lernmark, Å. , Pussinen, P. J. , & Liuba, P. (2018). HLA, infections and inflammation in early stages of atherosclerosis in children with type 1 diabetes. Acta Diabetologica, 55(1), 41–47. 10.1007/s00592-017-1063-1 29064046 PMC5794827

[cre2419-bib-0022] Rathnayake, N. , Gieselmann, D. R. , Heikkinen, A. M. , Tervahartiala, T. , & Sorsa, T. (2017). Salivary diagnostics‐point‐of‐care diagnostics of MMP‐8 in dentistry and medicine. Diagnostics (Basel, Switzerland), 7(1), 7. 10.3390/diagnostics7010007 PMC5373016 28117682

[cre2419-bib-0023] Schneeberger, E. E. , Citera, G. , Rodríguez Gil, G. , Granel, A. , Arturi, A. , Rosemffet, G. M. , Maldonado Cocco, J. A. , Berman, A. , Spindler, A. , & Morales, V. H. (2015). Clinical and immunogenetic characterization in psoriatic arthritis patients. Clinical Rheumatology, 34(8), 1413–1418. 25008283 10.1007/s10067-014-2739-3

[cre2419-bib-0024] Slots, J. (2017). Periodontitis: Facts, fallacies and the future. Periodontology 2000, 75(1), 7–23. 10.1111/prd.12221 28758294

[cre2419-bib-0025] Sorsa, T. , Alassiri, S. , Grigoriadis, A. , Räisänen, I. T. , Pärnänen, P. , Nwhator, S. O. , Gieselmann, D. R. , & Sakellari, D. (2020). Active MMP‐8 (aMMP‐8) as a grading and staging biomarker in the periodontitis classification. Diagnostics (Basel, Switzerland), 10(2), 61. 10.3390/diagnostics10020061 PMC7168924 31979091

[cre2419-bib-0026] Stein, J. M. , Machulla, H. K. , Smeets, R. , Lampert, F. , & Reichert, S. (2008). Human leukocyte antigen polymorphism in chronic and aggressive periodontitis among Caucasians: A meta‐analysis. Journal of Clinical Periodontology, 35(3), 183–192. 10.1111/j.1600-051X.2007.01189.x 18190553

[cre2419-bib-0027] Tonetti, M. S. , Greenwell, H. , & Kornman, K. S. (2018). Staging and grading of periodontitis: Framework and proposal of a new classification and case definition. Journal of Clinical Periodontology, 45(suppl 20), S149–S161. 10.1111/jcpe.12945 29926495

[cre2419-bib-0028] Tsuchiya, K. , Kimura, A. , Kondo, M. , Nishimura, Y. , & Sasazuki, T. (2001). Combination of HLA‐A and HLA class II alleles controls the susceptibility to rheumatoid arthritis. Tissue Antigens, 58(6), 395–401. 10.1034/j.1399-0039.2001.580608.x 11929590

[cre2419-bib-0029] Tuomainen, A. M. , Kormi, I. , Havulinna, A. S. , Tervahartiala, T. , Salomaa, V. , Sorsa, T. , & Pussinen, P. J. (2014). Serum tissue‐degrading proteinases and incident cardiovascular disease events. European Journal of Preventive Cardiology, 21(7), 806–812. 10.1177/2047487312465524 23079500

[cre2419-bib-0030] Vaterlaws, A. , Wolnizer, M. , Doran, T. , Bashir, H. , & Bhatia, K. (1989). HLA and nonspecific polyarthritis in the highlands of Papua New Guinea. Papua and New Guinea Medical Journal, 32(4), 263–268. 2626874

[cre2419-bib-0031] Vono, M. , Lin, A. , Norrby‐Teglund, A. , Koup, R. A. , Liang, F. , & Loré, K. (2017). Neutrophils acquire the capacity for antigen presentation to memory CD4^+^ T cells in vitro and ex vivo. Blood, 129(14), 1991–2001. 10.1182/blood-2016-10-744441 28143882 PMC5383872

[cre2419-bib-0032] Wen, G. , Zhang, C. , Chen, Q. , Luong, l. , Mustafa, A. , Ye, S. , & Xiao, Q. (2015). A novel role of matrix Metalloproteinase‐8 in macrophage differentiation and polarization. The Journal of Biological Chemistry, 290(31), 19158–19172. 10.1074/jbc.M114.634022 26092731 PMC4521038

