# Supplementary material for: Human leukocyte antigens are associated with salivary level of active MMP‐8
Source: Clin Exp Dent Res. 2021 Aug 27;7(5):833–9. doi: 10.1002/cre2.419 (PMC8543479; doi:10.1002/cre2.419)
Supplement: Supplementary file 1 — Table S1 Supporting information [file CRE2-7-833-s001.docx]

# **Supplementary tables**

Table 1. Associations of HLA-A types with the salivary levels of aMMP-8 in subjects with healthy periodontium or periodontitis

|  | Healthy (n = 86) | | | | | Periodontitis (n = 116) | | | | |
| --- | --- | --- | --- | --- | --- | --- | --- | --- | --- | --- |
|  | with A## | | without A## | | P | with A## | | without A## | | P |
|  | (n) | mean aMMP-8 (±SD) | (n) | mean aMMP-8 (±SD) |  | (n) | mean aMMP-8 (±SD) | (n) | mean aMMP-8 (±SD) |  |
| A01 | 24 | 168,8 (162,9) | 62 | 108,1 (115,1) | 0,10 | 30 | 204,0 (160,0) | 86 | 233,8 (191,3) | 0,64 |
| A02 | 39 | 191,7 (86,0) | 47 | 144,4 (159,0) | 0,50 | 59 | 205,7 (168,2) | 57 | 247,2 (197,4) | 0,31 |
| A03 | 26 | 103,9 (94,4) | 60 | 134,2 (145,2) | 0,60 | 32 | 230,0 (190,3) | 84 | 224,6 (182,0) | 0,99 |
| A11 | 9 | 49,2 (32,5) | 75 | 123,6 (119,2) | **0,048** | 10 | 394,2 (255,6) | 104 | 201,1 (146,1) | **0,016** |
| A23 | - | - | - | - | - | 7 | 182,8 (85,0) | 109 | 228,9 (188,0) | 0,89 |
| A24 | 17 | 158,5 (152,4) | 69 | 116,8 (126,5) | 0,24 | 20 | 261,9 (183,0) | 96 | 218,6 (183,0) | 0,25 |
| A26 | - | - | - | - | - | 9 | 179,7 (102,2) | 107 | 230,0 (188,6) | 0,73 |
| A29 | - | - | - | - | - | 6 | 212,7 (165,7) | 110 | 226,8 (185,1) | 1,0 |
| A30 | 5 | 118,9 (140,0) | 81 | 125,4 (132,5) | 0,79 | - | - | - | - | 0,55 |
| A31 | 6 | 144,9 (164,8) | 80 | 123,6 (130,5) | 0,88 | 5 | 340,5 (297,1) | 111 | 221,0 (177,2) | 0,45 |
| A32 | 7 | 166,0 (244,8) | 79 | 121,4 (119,4) | 0,63 | 15 | 266,8 (222,8) | 101 | 220,0 (177,5) | 0,52 |
| A33 | - | - | - | - | - | 5 | 176,2 (111,4) | 111 | 228,4 (186,2) | 0,74 |
| A68 | 10 | 116,0 (86,4) | 76 | 126,2 (137,4) | 0,64 | 10 | 153,5 (108,3) | 106 | 233,0 (188,0) | 0,28 |

- : not included if n<5; ##: the number of HLA-A type

Table 2. Associations of HLA-B types with the salivary levels of aMMP-8 in subjects with healthy periodontium or periodontitis

|  | Healthy (n = 86) | | | | | Periodontitis (n = 116) | | | | |
| --- | --- | --- | --- | --- | --- | --- | --- | --- | --- | --- |
|  | with B## | | without B## | | P | with B## | | without B## | | P |
|  | (n) | mean aMMP-8 (±SD) | (n) | mean aMMP-8 (±SD) |  | (n) | mean aMMP-8 (±SD) | (n) | mean aMMP-8 (±SD) |  |
| B07 | 19 | 83,9 (81,8) | 67 | 136,7 (141,5) | 0,15 | 33 | 188,4 (163,5) | 83 | 241,1 (189,8) | 0,16 |
| B08 | 12 | 141,2 (142,7) | 74 | 122,4 (131,1) | 0,50 | 16 | 241,7 (180,0) | 100 | 223,6 (184,9) | 0,54 |
| B13 | 7 | 136,9 (124,9) | 79 | 124,0 (133,4) | 0,71 | 8 | 325,7 (288,3) | 108 | 218,7 (173,1) | 0,51 |
| B14 | 7 | 96,3 (84,2) | 79 | 127,6 (135,6) | 0,72 | 7 | 170,4 (116,8) | 109 | 230,0 (186,8) | 0,56 |
| B15 | 14 | 156,0 (170,6) | 72 | 119,0 (123,9) | 0,57 | 10 | 257,8 (218,3) | 106 | 223,1 (180,8) | 0,76 |
| B18 | 8 | 195,8 (238,7) | 78 | 117,8 (116,3) | 0,87 | 14 | 234,1 (203,0) | 102 | 224,9 (181,8) | 0,98 |
| B27 | 7 | 94,1 (49,7) | 79 | 127,8 (136,9) | 0,87 | 11 | 175,5 (102,0) | 105 | 231,4 (189,6) | 0,61 |
| B35 | 20 | 132,8 (128,8) | 66 | 122,7 (133,9) | 0,74 | 21 | 248,9 (157,8) | 95 | 221,1 (189,2) | 0,20 |
| B37 | - | - | - | - | - | 5 | 289,9 (240,5) | 111 | 223,2 (181,5) | 0,48 |
| B38 | - | - | - | - | - | 6 | 184,5 (213,9) | 110 | 228,4 (182,6) | 0,37 |
| B39 | 5 | 120,7 (188,9) | 81 | 125,3 (129,5) | 0,34 | 7 | 204,0 (154,1) | 109 | 227,5 (185,8) | 0,93 |
| B40 | 14 | 142,0 (89,3) | 72 | 121,8 (139,2) | 0,09 | 8 | 149,4 (104,3) | 108 | 231,8 (187,2) | 0,32 |
| B44 | 20 | 128,9 (159,3) | 66 | 123,9 (124,1) | 0,86 | 28 | 261,1 (205,3) | 88 | 215,0 (175,9) | 0,34 |
| B49 | - | - | - | - | - | 9 | 263,4 (130,4) | 107 | 223,0 (187,5) | 0,21 |
| B51 | 9 | 171,5 (175,9) | 77 | 119,6 (126,4) | 0,34 | 11 | 193,0 (164,1) | 105 | 229,6 (185,8) | 0,57 |
| B55 | 5 | 76,4 (61,7) | 81 | 128,1 (134,9) | 0,51 | - | - | - | - | - |
| B57 | 6 | 85,2 (74,3) | 80 | 128,0 (135,3) | 0,58 | 11 | 209,6 (199,0) | 105 | 227,8 (182,8) | 0,63 |

-: not included if n<5; ##: the number of HLA-B type

Table 3. Associations of HLA-DRB types with the salivary levels of aMMP-8 in subjects with healthy periodontium or periodontitis

|  | Healthy (n = 86) | | | | | Periodontitis (n = 116) | | | | |
| --- | --- | --- | --- | --- | --- | --- | --- | --- | --- | --- |
|  | with ## | | without ## | | P | with ## | | without ## | | P |
|  | (n) | mean aMMP-8 (±SD) | (n) | mean aMMP-8 (±SD) |  | (n) | mean aMMP-8 (±SD) | (n) | mean aMMP-8 (±SD) |  |
| 01 | 21 | 128,2 (134,3) | 65 | 124,0 (132,4) | 0,88 | 19 | 246,7 (149,3) | 97 | 222,1 (189,9) | 0,24 |
| 03 | 15 | 135,4 (137,0) | 71 | 122,8 (131,9) | 0,66 | 20 | 259,1 (180,3) | 96 | 219,2 (184,4) | 0,22 |
| 04 | 19 | 133,3 (109,6) | 67 | 122,7 (134,4) | 0,30 | 35 | 252,5 (218,4) | 81 | 214,7 (166,5) | 0,68 |
| 07 | 23 | 89,4 (94,7) | 63 | 138,1 (141,8) | 0,07 | 31 | 231,8 (208,5) | 85 | 224,1 (174,8) | 0,80 |
| 08 | - | - | - | - | - | 8 | 139,6 (139,3) | 108 | 232,5 (185,3) | 0,13 |
| 11 | 20 | 111,6 (111,3) | 66 | 129,1 (138,3) | 0,63 | 28 | 249,3 (209,4) | 88 | 218,7 (175,2) | 0,63 |
| 12 | 6 | 122,8 (108,4) | 80 | 125,2 (134,3) | 0,77 | 5 | 259,0 (143,1) | 111 | 224,6 (185,5) | 0,43 |
| 13 | 22 | 135,0 (135,2) | 64 | 121,6 (131,9) | 0,59 | 26 | 191,4 (147,6) | 90 | 236,1 (192,3) | 0,43 |
| 14 | 11 | 152,1 (201,6) | 75 | 121,0 (120,1) | 0,92 | 7 | 312,4 (210,1) | 109 | 220 (181,4) | 0,17 |
| 15 | 16 | 171,4 (199,0) | 70 | 114,5 (110,8) | 0,29 | 27 | 184,4 (170,9) | 89 | 238,8 (186,3) | 0,12 |

-: not included if n<5; ##: the number of HLA-DBR type
